# Evaluation of Green and Biobased Solvent Systems for the Extraction of β-Carotene and Lipids from *Rhodosporidium toruloides*

**DOI:** 10.1021/acsomega.4c10851

**Published:** 2025-01-27

**Authors:** Vanessa Buchweitz, Kilian Dauti, Ahmad Alhadid, Mirjana Minceva

**Affiliations:** Biothermodynamics, TUM School of Life Sciences, Technical University of Munich, Freising 85354, Germany

## Abstract

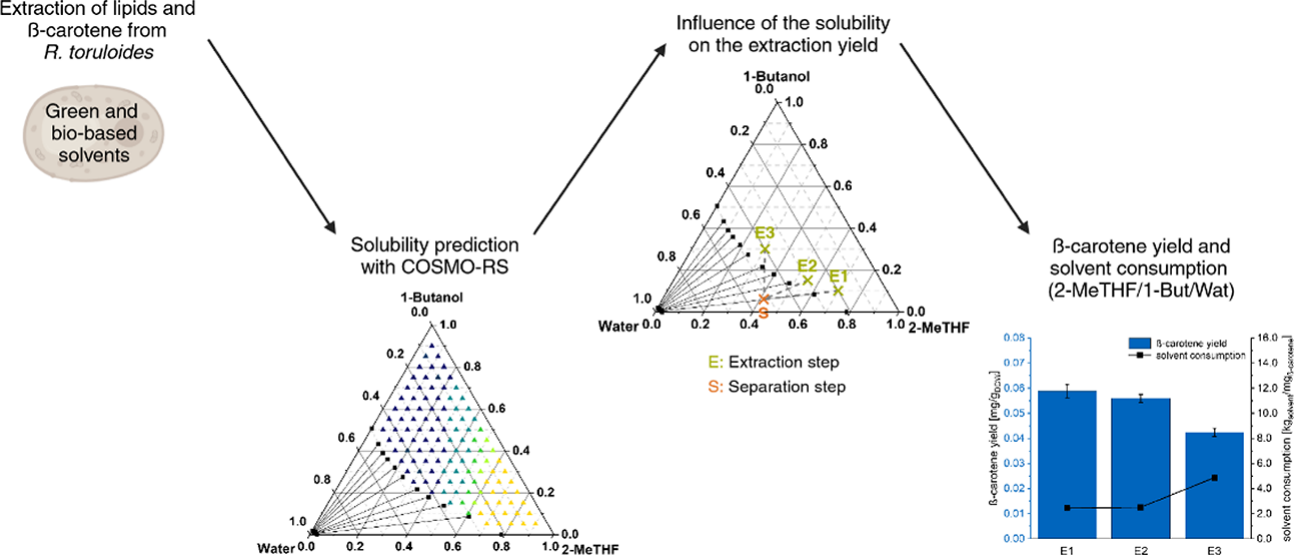

Oleaginous microorganisms are promising for the biotechnological production of valuable hydrophobic bioactive components. For an environmentally friendly extraction, we evaluated a two-step process for β-carotene and lipid isolation from wet *Rhodosporidium toruloides* biomass using biphasic green and biobased solvent systems: 2-methyl tetrahydrofuran (2-MeTHF) or cyclopentyl-methyl-ether (CPME) with ethanol or 1-butanol and water. Initially, components were extracted with a single-phase solvent mixture, followed by separating hydrophobic target components from polar impurities via phase separation. We employed the Conductor-like Screening Model for Real Solvents (COSMO-RS) to predict the solubility of β-carotene and select compositions with higher solubility. Our study highlights the potential of these solvent systems for extracting hydrophobic components and the importance of understanding the system’s liquid–liquid equilibria for effective process design. We present a framework for evaluating new solvent systems for extracting hydrophobic bioactive compounds by demonstrating the impact of solvent composition selection on extraction yields and solvent consumption.

## 1 Introduction

The biotechnological production of oleochemicals and hydrophobic components has gained interest due to limited natural resources and declining petroleum deposits.^1,2^ Oleaginous microorganisms are a promising sustainable option for producing hydrophobic components used in the pharmaceutical, food, and feed industries and for biofuels.^3−5^

To ensure that biotechnologically produced products are competitive in the market, a low-energy and low-cost extraction process is required.^6^ As the hydrophobic components are produced intracellularly, the cells must be lysed before extraction.^7^ Drying the biomass before extraction is efficient but requires an energy-consuming drying step. Notably, drying processes consume 17.7% of the total energy consumption of industries. This has motivated the development of extraction methods from wet biomass with high recovery rates of hydrophobic target components to achieve more energy-efficient processes that could be used in the industrial field.^8,9^ A challenge for the extraction of cellular lipids from wet biomass is the strong interactions between lipids and cell biopolymers like proteins and polysaccharides.^10^ When using single nonpolar solvents, such as chloroform (TCM) or hexane (Hex), for the extraction of lipids from wet biomass, low extraction yields are obtained since the water contained in the biomass hinders the direct contact with the solvent and the dissolution of lipids.^11^ Therefore, a combination of both polar and nonpolar solvents is necessary to disrupt these molecular interactions and extract both polar and nonpolar lipids.^10^ Hence, a two-step extraction process with biphasic ternary solvent systems is used, consisting of an extraction step with a solvent system composition within the single-phase region, followed by a separation step within the biphasic region to separate the extracted hydrophobic and hydrophilic components. The most common methods using this two-step extraction process for analytical purposes of extracting lipids from biological samples are the Folch method^12^ and the Bligh and Dyer^13^ method. These methods utilize a biphasic solvent system with chloroform, methanol (MeOH), and water (Wat) but different solvent ratios. Silve et al.^6^ and Gorte et al.^14^ applied the principle of these methods to the extraction of lipids from oleaginous microorganisms with the solvent system hexane + ethanol (EtOH) + water and increased the mixing time during the extraction step.

In recent years, significant efforts have been directed toward identifying green and biobased solvent systems as sustainable alternatives to harmful conventional solvents for extracting hydrophobic compounds, mainly oils, from oleaginous microorganisms.^15^ Green solvents are solvents that meet most of the 12 principles of green chemistry described by Anastas and Warner^16^ in 1998, which aim for less toxic, environmentally friendlier, less hazardous, and less energy-consuming processes. Biobased solvents represent a group of green solvents derived from renewable sources.^17,18^

Mussagy et al.^19^ applied the green and biobased solvent systems ethanol + (ethyl acetate or limonene) + water to extract astaxanthin and β-carotene from both wet and dry biomass of the yeast *Phaffia rhodozyma*, with a heat-assisted extraction. The highest yield was achieved from wet biomass with pure solvent ethanol, but the extraction yields obtained with the ternary solvent system were higher for wet biomass than for dried biomass. For the extraction of lipids and carotenoids from wet biomass from the oleaginous yeast *Rhodotorula glutinis*, Mussagy et al.^20,21^ examined the green and biobased solvent systems ethanol + (ethyl lactate or ethyl acetate) + water also using a heat-assisted extraction. They found that the ternary solvent systems achieved higher recovery yields than the pure solvents and traditionally used methods by Folch and Bligh and Dyer. De Jesus et al.^11,22^ studied both single green solvents, 2-MeTHF and CPME, and the green solvent systems (2-MeTHF or CPME) + isoamyl alcohol + water for the extraction of lipids from the oleaginous microalgae *Chlorella pyrenoidosa*. In this work, higher extraction yields were also obtained when two solvents of different polarities instead of a single nonpolar solvent were added to the wet biomass. These observations confirm the hypothesis that a second polar solvent is needed to interrupt the molecular interactions between the lipids and cell biopolymers. The above-referred works experimentally evaluated the potential of the green and biobased solvent systems studied. The system compositions for the experiments were arbitrarily selected or determined after an extensive experimental screening.

For the design of the extraction process and selection of the solvent system composition for the two steps, in the single-phase region for the extraction step and in the biphasic region for the separation step, knowledge of the liquid–liquid equilibria of the solvent systems is essential. The experimental screening of different solvent system compositions involves very time-consuming experiments. Therefore, predictive thermodynamic models such as the Conductor-like Screening Model for Real Solvents (COSMO-RS) can be applied to predict the solubility of the targeted components within the single-phase region. The COSMO-RS model has been shown to provide reliable predictions for the thermodynamic properties of multicomponent systems, requiring only the molecular structure of components to predict the activity coefficients of components.^23−26^

In this work, the green and biobased solvent systems 2-MeTHF + (ethanol or 1-butanol) + water and CPME + (ethanol or 1-butanol) + water were evaluated as a sustainable alternative to conventional extraction methods that use harmful solvents like chloroform and hexane for the extraction of β-carotene and cellular lipids from wet *Rhodosporidium toruloides* (*R. toruloides*) biomass. This oleaginous yeast can accumulate lipids intracellularly to over 50% of their dry cell weight (DCW) and produces various valuable bioactive compounds, including β-carotene, a lipid-soluble antioxidant, and colorant widely used in medicine, health products, cosmetics, and the food and feed industry, with an expected market value of USD 780 million by 2027.^27−29^ Biotechnologically produced β-carotene is considered natural and therefore preferable over synthetically produced β-carotene due to health benefits and higher consumer acceptance.^30^ Due to the similar fatty acid composition of lipids produced by *R. toruloides* to the composition of cocoa butter, they present a possible sustainable substitution option.^31^ To show the importance of the selection of solvent system compositions for both process steps, its influence on the extraction yields and solvent consumption was evaluated. To avoid time-consuming experiments and an arbitrary selection of solvent compositions, the solubility of β-carotene within the single-phase region of all four green and biobased solvent systems was predicted with COSMO-RS and used to select solvent system compositions for the experimental evaluation of the extraction process performance. Additionally, the extraction yields obtained with the green and biobased solvent systems were compared to yields achieved with the abovementioned methods using conventional organic solvents.^12−14^

## 2 Materials and Methods

### 2.1 Chemicals

2-Methyltetrahydrofuran (2-MeTHF; 99.9%), cyclopentyl-methyl-ether (CPME; >99%), chloroform (>99%), and hexane (≥99%) were acquired from Merck (Merck KGaA, Darmstadt, Germany). Ethanol (>99.9%), 1-butanol (99.86%), tetrahydrofuran (THF, 99.9%), tridecanoic acid (≥99.0%), nonadecanoic acid (≥98.0%), cis-10-nonadecenoic acid (≥99%), and fatty acid methyl ester (F.A.M.E) Mix (C8–C24, > 99%) were bought from Sigma-Aldrich (Sigma-Aldrich, St. Louis, USA), while water (>99.9%), methanol (≥99.9%), and β-carotene (>98.0%) were purchased from VWR Chemicals (VWR International, Radnor, USA). A detailed composition of the FAME Mix can be found in Table S1.

### 2.2 Solubility Measurements

β-Carotene solubility was measured at 298.15 K in the pure solvents 2-MeTHF, CPME, ethanol, and 1-butanol and preselected compositions in the single-phase region of four green and biobased solvent systems: 2-MeTHF + (ethanol or 1-butanol) + water and CPME + (ethanol or 1-butanol) + water. The liquid–liquid equilibrium data of these solvent systems were taken from our previous work.^32^ The system compositions of each solvent system at which the solubility was measured are listed in Tables S2 and S3, which were selected to cover a range of β-carotene solubilities.

Each solvent system composition was prepared by weighing the solvents on an analytical balance (SECURA124–1S, Sartorius AG, Germany) with a repeatability of 0.0001 g. An excess amount of β-carotene was added to the pure solvent or solvent mixture until an oversaturated solution with a persisting precipitate was obtained. Then, the samples were mixed with a temperature-controlled thermomixer (ThermoMixer F2.0, Eppendorf SE, Germany) protected from sunlight for 30 min, followed by centrifugation (Micro Star 12 Microcentrifuge, VWR International GmbH, USA) at 13500 rpm for 10 min. The β-carotene concentration of the diluted supernatant was determined with a UV/vis spectrophotometer (Specord 50 plus, Analytik Jena GmbH+Co.KG, Germany) at the absorption maximum of 453.5 nm. β-Carotene is an unstable component prone to oxidation;^33^ therefore, it is difficult to avoid β-carotene oxidation during the solubility measurements. Thus, the measured data can be considered apparent rather than the equilibrium solubility data.^34^ The loss of β-carotene during solubility measurements after 270 min is presented in Table S4.

### 2.3 Solubility Prediction with COSMO-RS

The solubility of β-carotene in a solvent system is calculated as follows:^35^
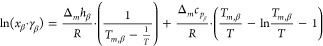
1where *x*_β_ and *γ*_β_ are the mole fraction and activity coefficient of β-carotene in the liquid phase, respectively; Δ_*m*_*h*_β_ and *T*_*m*,β_ are the melting enthalpy and temperature of pure β-carotene, respectively; Δ_*m*_*c*_*pi*_ is the difference between the heat capacity of β-carotene in the solid and liquid states at its melting temperature; *R* is the ideal gas constant; and *T* is the solution temperature. Due to the thermal instability of β-carotene, the melting properties are unavailable, and the solubility cannot be directly calculated by COSMO-RS using eq 1. To overcome this issue, the approach proposed by Vilas-Boas et al.^36^ was applied, which uses an experimentally measured solubility as a reference point () to estimate the solubility of the target component in another solvent or solvent mixture. The ratio between the β-carotene solubility in any particular solvent or solvent mixture and that at the reference experimental solubility point can be calculated as follows:
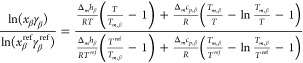
2where  is the β-carotene activity coefficient in the liquid phase at the temperature and composition of the reference solubility point. At a constant temperature, eq 2 can be reduced to the following relation:
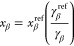
3

COSMO-RS was used to calculate the activity coefficient of components in the liquid phase from the molecular structure.^37,38^ COSMOconf (ver. 4.3, Dassault Systèmes, France) was used to generate the molecular conformations of components. The molecular geometry optimization and screening charge density calculations were performed by using Turbomole (version 6.6, TURBOMOLE GmbH, Germany). The DFT calculations were performed by using def-TZVP basis sets. COSMOtherm 19 (Version 19.0.5, Dassault Systèmes, France) was used to calculate the activity coefficients using the BP_TZVP_19.ctd parameter set.

### 2.4 Biomass Specification

*Rhodosporidium toruloides* IFO0559 and IFO0880 (corresponding to ATCC10788/CBS 14 and ATCC10657/CBS 349, respectively) were cultivated by the Werner Siemens-Chair of Synthetic Biotechnology (Technical University of Munich) with a DASgip System (Eppendorf SE, Hamburg, Germany) with Yeast Nitrogen Base (YNB) medium (Carl Roth GmbH + Co. KG, Karlsruhe, Germany) at 301.15 K. After 80 h, 2.5 l with an optical density (OD) of 67.8 was reached, and the biomass was concentrated to 600 mL. The biomass’s dry cell weight (DCW) was determined through lyophilization (DCW = 0.13), and the method is described in the Supporting Information. The cells were disrupted with a high-pressure homogenizer, Emulsiflex B15 (Avestin Europe GmbH, Mannheim, Germany) at 2000 bar, and cell disruption was verified with a light microscope equipped with a Thoma hemocytometer from Brand (BRAND GMBH + CO KG, Wertheim, Germany).

### 2.5 Hydrophobic Component Extraction

**Figure 1:**
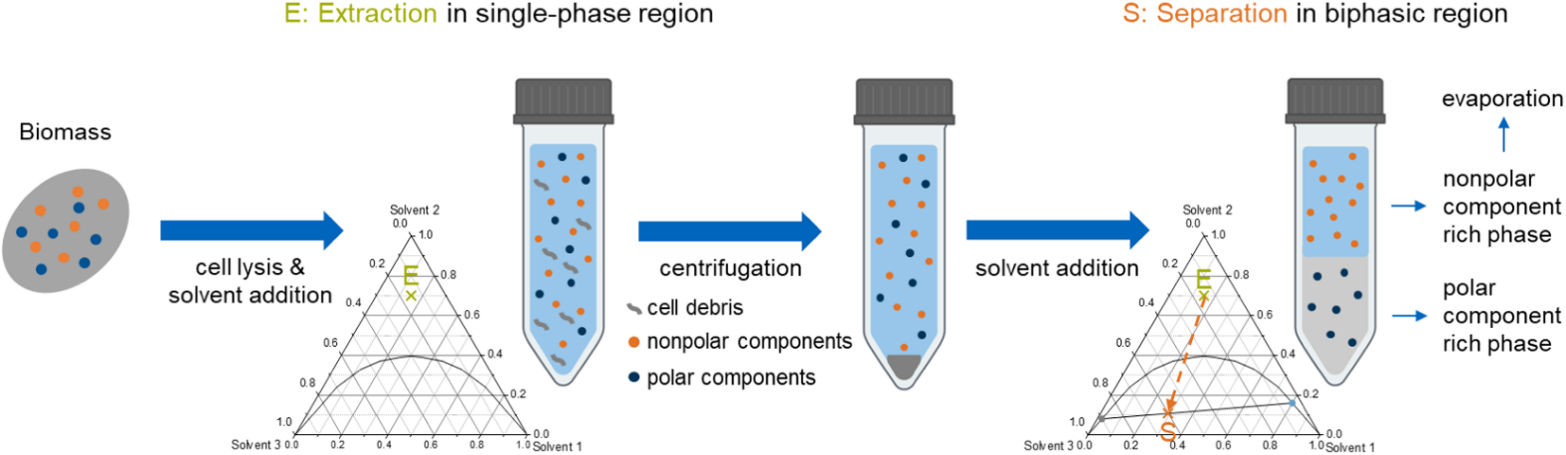
Scheme of the process for the extraction of β-carotene and lipids from the oleaginous yeast *R. toruloides*.

For the extraction of cellular lipids and β-carotene from the wet biomass of *R. toruloides*, the two-step method used by Silve et al.^6^ and Gorte et al.^14^ was applied. The extraction process consists of two steps: an extraction step within the single-phase region of a biphasic solvent system phase diagram (point E in Figure 1) where the hydrophobic and hydrophilic components of the wet biomass were extracted, followed by a separation step within the biphasic region (point S in Figure 1) to separate the hydrophobic components in the solvent-rich phase from the hydrophilic ones in the water-rich phase. The method is presented in Figure 1 and is described in detail in the Supporting Information.

In this work, instead of the conventional solvent system hexane + ethanol + water, the green and biobased solvent systems 2-MeTHF + (ethanol or 1-butanol) + water and CPME + (ethanol +1-butanol) + water were used. Different solvent system compositions for the extraction point (E) and separation point (S) were selected for the extraction experiments to study the influence of the compositions on the extraction yields and solvent consumption. They are provided in Table S5. Each extraction experiment was performed in triplicate, and blank extractions without biomass were used to exclude artifacts.

The total extraction yield was determined gravimetrically after evaporating the solvent in “gram extract per gram DCW” (eqs S1 and S2), and the β-carotene yield was determined, as described in Section 2.2, and presented in “milligram β-carotene per gram DCW” (eq S3). Furthermore, the composition of the fatty acid methyl esters of the extracted lipids was determined by gas chromatography, and the method is detailed in the Supporting Information. A statistical analysis was performed for all experimental results, which is described in the Supporting Information.

## 3 Results and Discussion

### 3.1 Experimental Solubility Measurements

**Figure 2:**
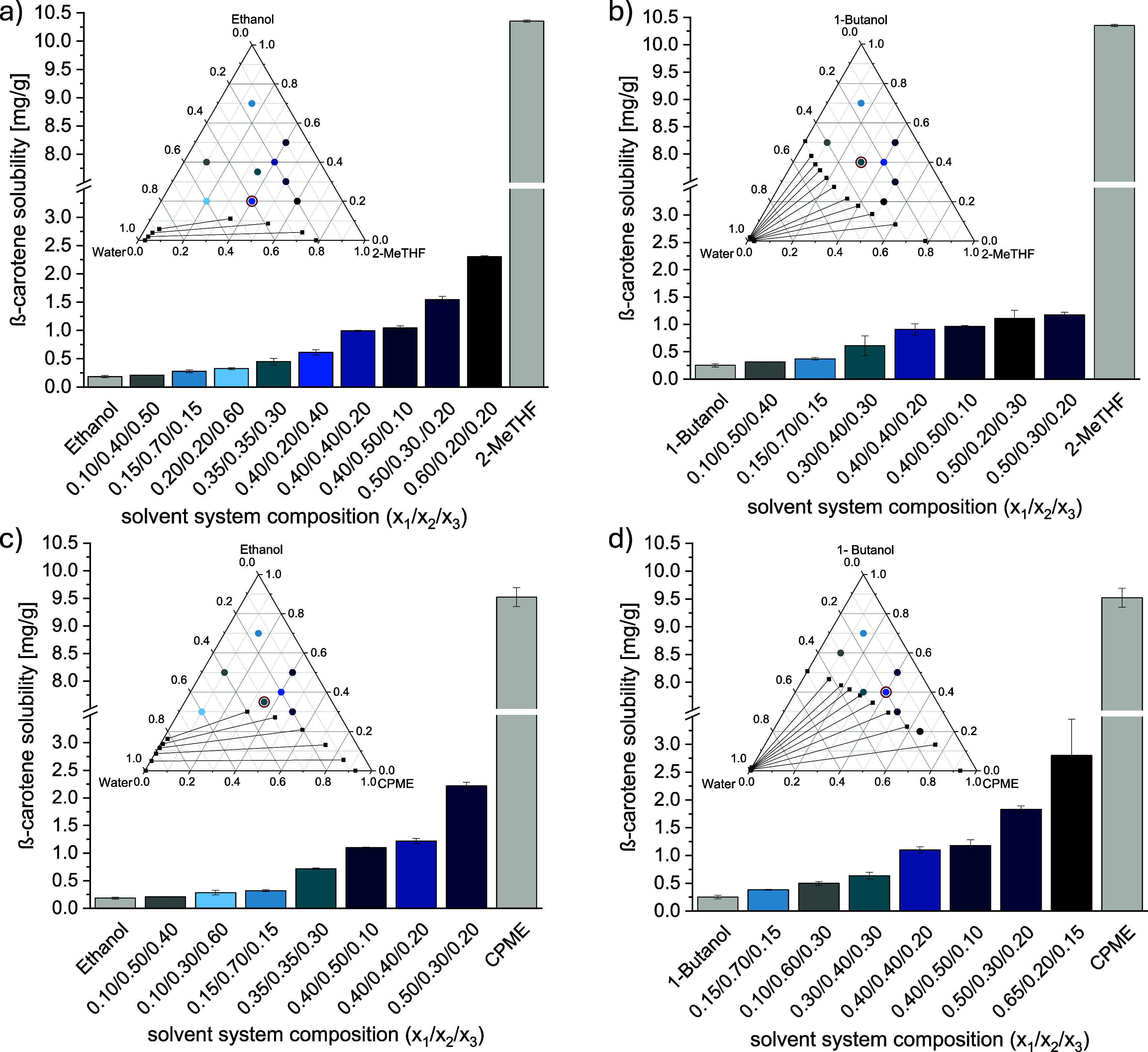
Experimental solubility of β-carotene in pure solvents 2-MeTHF, CPME, ethanol, and 1-butanol and different solvent system compositions (mole fractions) of the systems: (a) 2-MeTHF (1) + ethanol (2) + water (3); (b) 2-MeTHF (1) + 1-butanol (2) + water (3); (c) CPME (1) + ethanol (2) + water (3); (d) CPME (1) + 1-butanol (2) + water (3); with red ○ as the solvent system compositions used as a reference for the solubility predictions.

The solubility of β-carotene was measured in selected compositions of the four solvent systems within the single-phase region and in pure 2-MeTHF, CPME, ethanol, and 1-butanol. The results are listed in Figure 2.

As seen in Figure 2, β-carotene had a low solubility in both alcohols. In contrast, the solubilities in 2-MeTHF (10.35 mg_β-carotene_/g) and CPME (9.52 mg_β-carotene_/g) were similar and significantly higher than in the polar alcohols (ethanol: 0.18 mg_β-carotene_/g, 1-butanol: 0.25 mg_β-carotene_/g). β-Carotene is a hydrophobic component (octanol/water coefficient log(P_ow_) = 17.6^39^) that is insoluble in water.^40^ Accordingly, β-carotene is significantly more soluble in nonpolar solvents (2-MeTHF: log(P_ow_) = 1.85,^41^ and CPME: log(P_ow_) = 1.59^42^ than in polar solvents (ethanol: log(P_ow_) = −0.30, and 1-butanol: log(P_ow_) = 0.84).^43^ The apparent solubility of β-carotene in solvent systems rich in water and alcohol was lower than that in solvent systems rich in 2-MeTHF or CPME. Thus, the solubility of β-carotene in the solvent systems was in agreement with its solubility in pure solvents, as shown in Figure 2. In all experimentally evaluated systems, the highest solubility was achieved with the solvent system CPME + 1-butanol + water at a composition of 0.65/0.20/0.15 (mol/mol/mol, Figure 2) with 2.80 mg_β-carotene_/g, which was the composition with the highest mole fraction of a nonpolar solvent. It stands out that when comparing the results for the solvent composition with the highest mole fraction of alcohols (0.15/0.70/0.15 mol/mol/mol, Figure 2), the β-carotene solubility was significantly higher in systems containing 1-butanol compared to systems containing ethanol, attributed to the higher solubility of β-carotene in 1-butanol than in ethanol (gray colored bars in Figure 2). In contrast, this trend was reversed at a lower mole fraction of the alcohol. For instance, the solubility of β-carotene in the systems with the solvent system composition of 0.50/0.30/0.20 (mol/mol/mol, Figure 2) was higher in systems containing ethanol than those containing 1-butanol. Additionally, the β-carotene solubility at the same solvent system composition was significantly higher in both solvent systems containing CPME than in those containing 2-MeTHF.

### 3.2 Solubility Predictions with COSMO-RS

**Figure 3:**
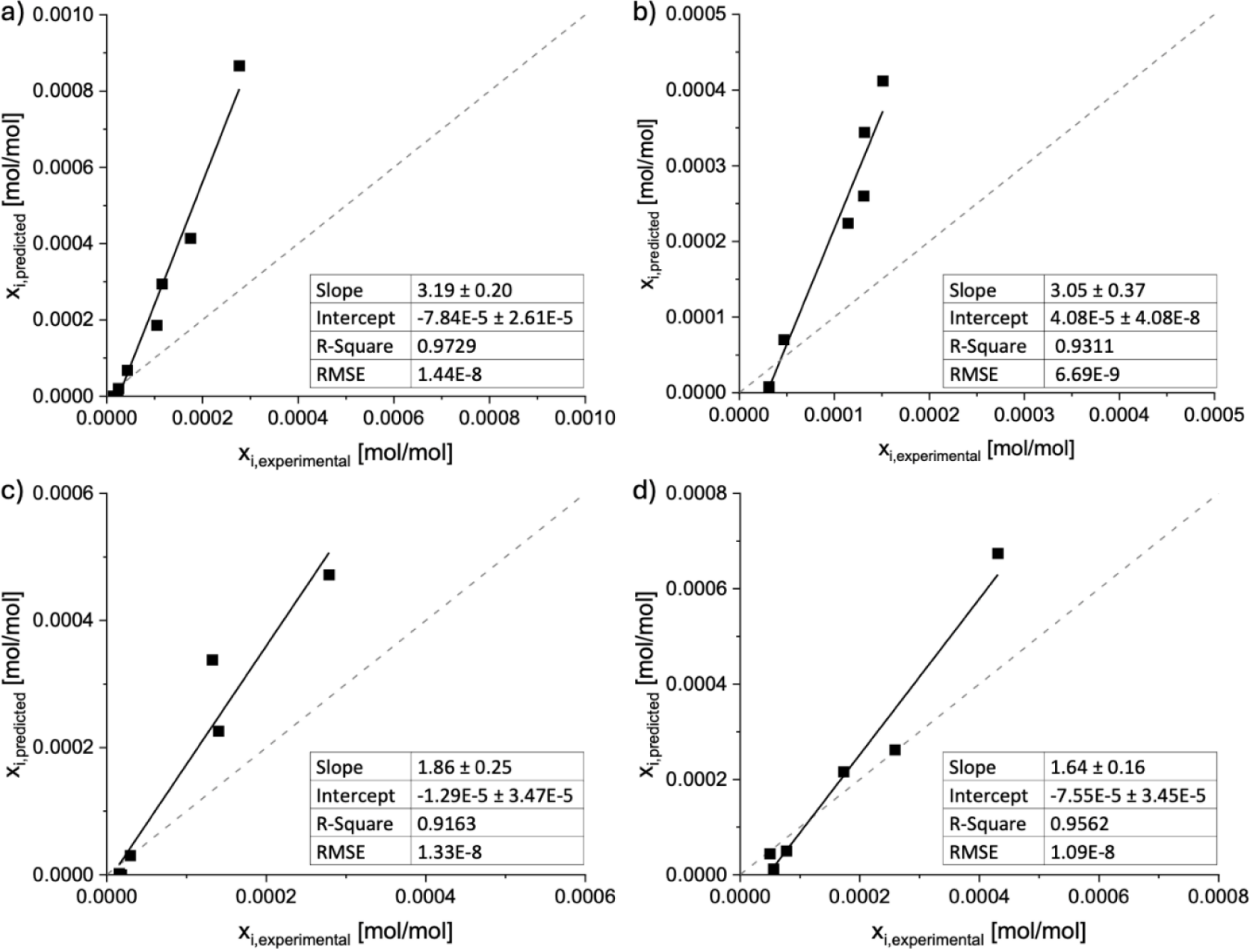
A comparison between experimental data and COSMO-RS predictions of β-carotene solubility in (a) 2-MeTHF + ethanol + water; (b) 2-MeTHF + 1-butanol + water; (c) CPME + ethanol + water; and (d) CPME + 1-butanol + water.

The solubility of β-carotene in various solvent mixtures within the single-phase region of the four solvent systems was predicted with COSMO-RS to reduce the experimental efforts needed to screen different compositions of the solvent systems. An experimental solubility point of each solvent system was selected as the reference point (). The activity coefficients of β-carotene in the liquid phase at the solvent system composition of interest (γ_β_) and reference composition () were calculated using COSMO-RS. Accordingly, the β-carotene solubility in the solvent system composition of interest (*x*_β_) was calculated using eq 3. For each solvent system, a system composition from the experimental data presented in Figure 2 (marked with circle open) within the medium range of measured β-carotene solubility was selected as . The selected compositions are marked in Figure 2 and are listed in Table S7. Figure 3 shows a comparison between the experimental and predicted β-carotene solubilities for all four solvent systems.

As seen in Figure 3, COSMO-RS overestimated the solubility of β-carotene in all solvent systems. Nevertheless, a good qualitative prediction was obtained with R^2^ values above 0.91 and low root-mean-square error (RMSE) for all solvent systems. Therefore, COSMO-RS can be used to estimate the apparent solubility of β-carotene in the solvent system compositions within the entire single-phase region, utilizing the linear correlation shown in Figure 3. Although the selection of the reference point influenced the linear correlation parameters, the calculated β-carotene solubility was always the same regardless of the selected reference point (Table S6). Additionally, the solubility of β-carotene within the pure solvents was calculated and compared to the experimentally measured one, which is presented in Table S7.

**Figure 4:**
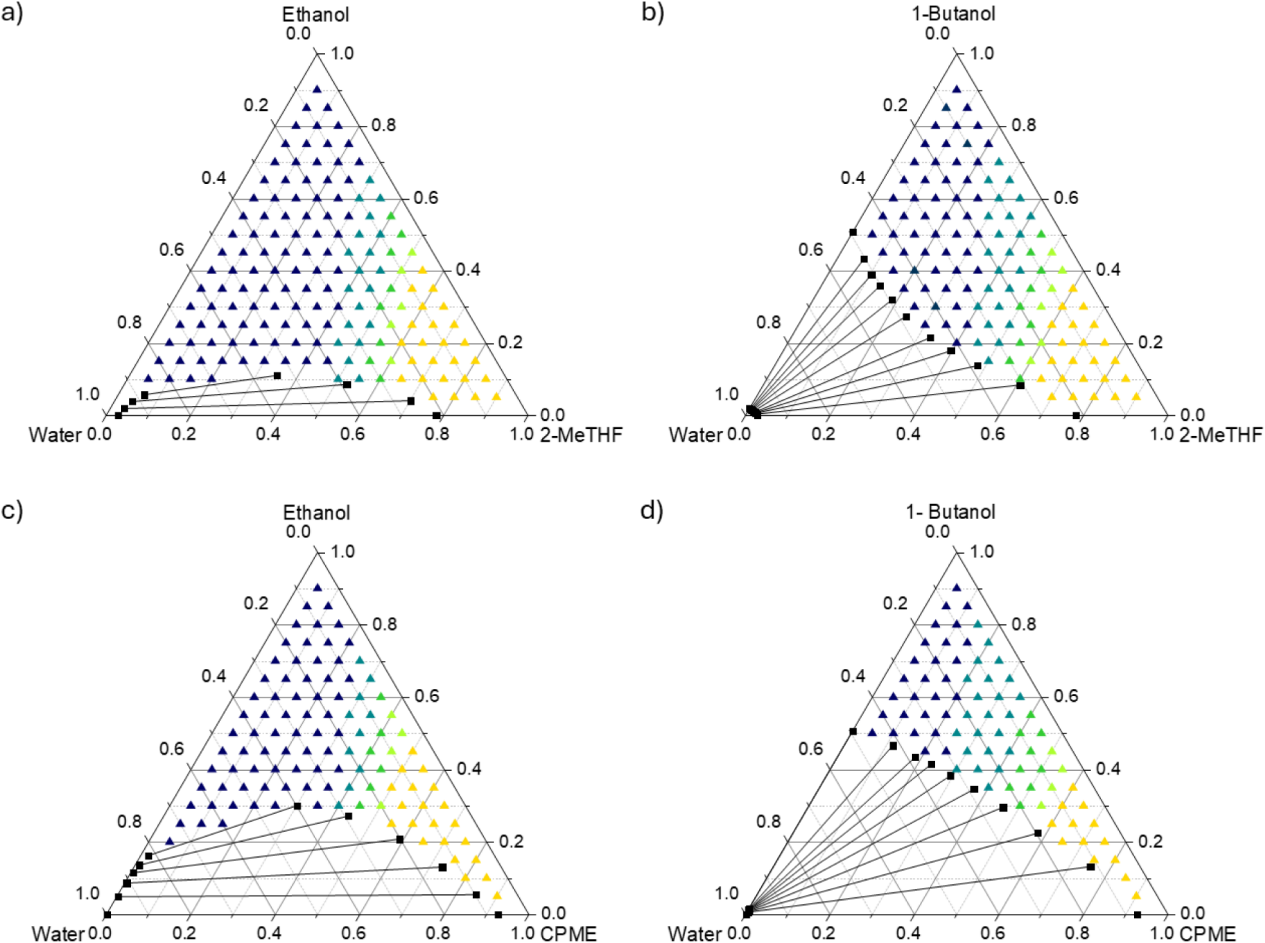
Ternary diagrams of the solvent systems (a) 2-MeTHF (1) + ethanol (2) + water (3); (b) 2-MeTHF (1) + 1-butanol (2) + water (3); (c) CPME (1) + ethanol (2) + water (3); (d) CPME (1) + 1-butanol (2) + water (3) with predicted solubility of β-carotene within the single-phase region; (dark blue ▲) < 0.6 mg/g; (light blue ▲) 0.6–1.2 mg/g; (dark green ▲) 1.2–1.8 mg/g; (light green ▲) 1.8–2.4 mg/g; (yellow ▲) > 2.4 mg/g.

The calculated β-carotene solubility in solvent mixtures with composition within the entire single-phase region of the solvent systems 2-MeTHF + (ethanol or 1-butanol) + water and CPME + (ethanol or 1-butanol) + water is presented in Figure 4. Five solubility ranges were defined and presented with different colors in the ternary diagrams in Figure 4.

As seen in Figure 4, the predicted solubility of β-carotene in solvent systems rich in water and alcohols was the lowest for all systems, as indicated by the dark blue triangles (dark blue ▲). Moreover, the region for the lowest solubility (dark blue ▲) was larger for the systems containing ethanol (Figure 4a and c) compared to the ones containing 1-butanol (Figure 4b and d), which agreed with the lower solubility of β-carotene in the pure alcohols shown in Figure 2. These findings also corresponded with the smaller miscibility gap of the systems containing ethanol compared to the ones with 1-butanol, which have a bigger miscibility gap and a lower phase that mostly consisted of water. To the same extent, the region with the highest solubility (yellow ▲) for the systems containing CPME was smaller than for those with 2-MeTHF, attributed to the higher mutual solubility (i.e., smaller miscibility gap) of 2-MeTHF with water compared to CPME.^32^ For both systems containing 2-MeTHF (Figure 4a,b), a solubility higher than 2.4 mg/g was obtained when the water mole fraction was ≤0.25 and the alcohol mole fraction was ≤0.40. On the other hand, a solubility higher than 2.4 mg/g was achieved with a water mole fraction ≤0.20 and an alcohol mole fraction ≤0.45 for the systems containing CPME (Figure 4c,d).

### 3.3 Extraction of β-Carotene and Lipids from *R. toruloides*: Extraction Yield and Solvent Consumption

**Figure 5:**
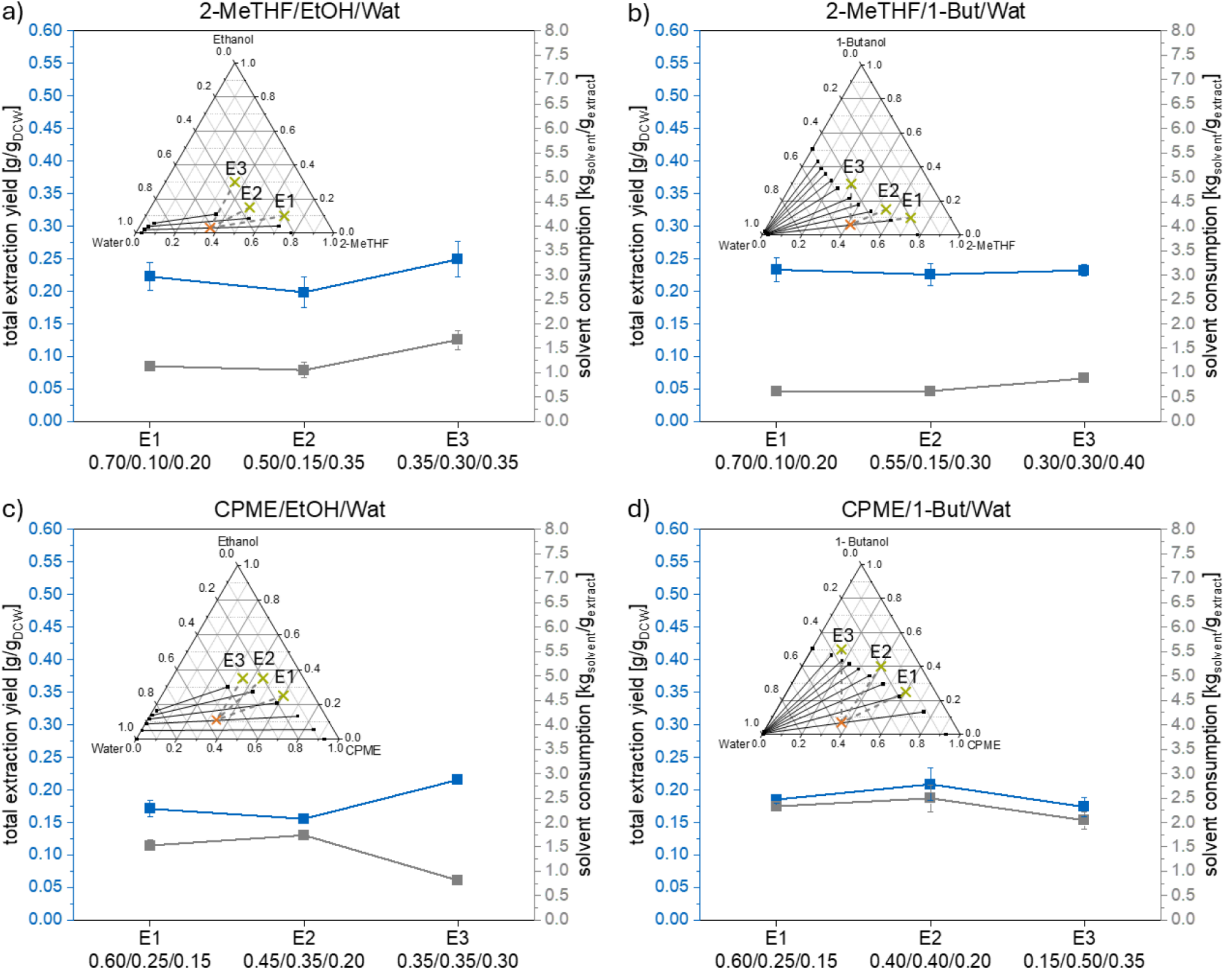
Total extraction yield (blue) and solvent consumption (gray) for 1 g extract for the extraction experiments (E1–E3) for the solvent systems: (a) 2-MeTHF + ethanol + water; (b) 2-MeTHF + 1-butanol + water; (c) CPME + ethanol + water; and (d) CPME + 1-butanol + water with the same solvent composition for the separation point (orange x) for each solvent system.

**Figure 6:**
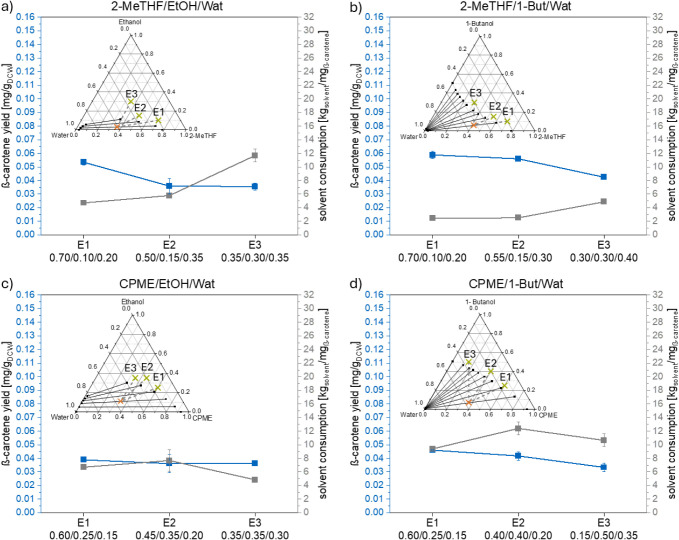
β-Carotene yield (blue) and solvent consumption (gray) for 1 mg of β-carotene for the extraction experiments (E1–E3) for the solvent systems (a) 2-MeTHF + ethanol + water; (b) 2-MeTHF + 1-butanol + water; (c) CPME + ethanol + water; and (d) CPME + 1-butanol + water with the same solvent composition for the separation point (orange x) for each solvent system.

The influence of the composition of the solvent system on the extraction yield of both cellular lipids and β-carotene from wet biomass and the respective solvent consumption was determined by selecting three compositions (points E1–E3 in the ternary diagrams in Figures 5 and 6) of the solvent systems having different β-carotene solubility values for the extraction step. On the other hand, the same solvent system composition from the biphasic region was selected for the separation step (marked with an orange cross in the ternary diagrams in Figures 5 and 6). This was done to ensure that the differences in the obtained extraction yields were due to only the difference in the composition of the solvent system used for the extraction step. The system compositions (E1–E3) in mole fractions and the corresponding β-carotene solubility calculated using the approach described in Section 2.3 are provided in Table S8.

In Figure 5 the total extraction yields and respective solvent consumptions for the three selected systems are presented. The total extraction yield was calculated by using the mass of extract recovered in the upper phase of the system used in the separation step, marked with an orange cross in Figures 5 and 6, after the evaporation of the solvents. In agreement with Jesus et al.,^11,22^ Silve et al.,^6^ and Gorte et al.^14^ no lipids were found in the lower polar solvent-rich phase for all studied solvent systems. Furthermore, the required amount of solvent to obtain the extract is another important factor in designing an extraction process. Therefore, the solvent consumption per 1 g of total extract and 1 mg of β-carotene were calculated, as described in the Supporting Information. Additionally, the needed amounts of the individual solvents for each experiment are presented in Figures S2 and S3.

As seen in Figure 5, for all studied systems, the different solvent system compositions selected for the extraction step had no significant influence on the total extraction yield when the composition for the separation step remained the same. The achieved yields were similar and within the range of 0.20 and 0.25 g/g_DCW_. The systems containing 2-MeTHF resulted in a slightly higher total extraction yield (mean: 0.23 g/g_DCW_) than those with CPME (mean: 0.18 g/g_DCW_). This might be explained by the higher mole fractions of ethanol or 1-butanol in the solvent systems containing CPME.

Figure 5 shows that the selection of solvent system compositions for the extraction and separation steps greatly influences the solvent consumption to obtain 1 g of extract. Considering that the extraction yield obtained in the different extraction experiments (E1–E3) did not differ significantly, the observed differences in the solvent consumption result mainly from the differences in the solvent system compositions used in the extraction and separation step. This can be explained by the different amounts of solvents needed to be added to the wet biomass (0.87 g of water per 1 g of biomass) to reach the solvent system composition for the extraction step, resulting in a different solid–liquid ratio for each extraction experiment (Table S5). The higher the water mole fraction in the system selected for the extraction step, the less solvent 1 (2-MeTHF/CPME) and solvent 2 (ethanol/1-butanol) needed to be added to the biomass in the extraction step. Additionally, due to the same solvent system composition for the separation step, the amount of solvent needed to be added to the single-phase solvent mixture used in extraction (after removing the cell debris with centrifugation; see Figure 1) varies for each experiment (E1–E3) of each solvent system. The solvent system composition for the separation step in the biphasic region of the solvent system has a lower mole fraction of solvent 2 (ethanol/1-butanol) than for the extraction step. Hence, an additional amount of solvent 1 (2-MeTHF/CPME) and solvent 3 (water) must be added to the system to reach the selected biphasic solvent system composition for the separation step. Overall, the lowest solvent consumption per 1 g extract of all solvent systems with the selected conditions was with the solvent system 2-MeTHF + 1-butanol + water and the experiment E1. This can be explained by the high total extraction yield, a low mole fraction of 1-butanol in the solvent system composition for the extraction step, and the small distance between the compositions for the extraction (E) and separation (S) steps. The further the distance between the solvent system composition for the extraction (E) and separation (S) step, the more solvents must be added to reach the composition for the separation step (S).

In Figure 6, the achieved β-carotene extraction yields and respective solvent consumption per 1 mg of extracted β-carotene for the studied systems are presented. For all solvent systems, the highest yield was obtained with the solvent system composition with the highest predicted β-carotene solubility (E1). The highest β-carotene yield of all four systems was achieved with the solvent system 2-MeTHF + 1-butanol + water (0.06 mg_β-carotene_/mg_DCW_). When using the solvent system compositions with a high predicted β-carotene solubility (E1) for the extraction step, the β-carotene yield was significantly higher than the β-carotene yield obtained at the solvent system composition with a low predicted solubility E3 (Table S8) for the solvent systems 2-MeTHF + (ethanol or 1-butanol) + water and CPME + 1-butanol + water. Hence, it can be stated that the solvent system composition selected for the extraction step has an influence on the β-carotene yield and a composition with a high solubility of the target component should be chosen for the extraction process.

As seen in Figure 6, for the extraction of 1 mg of β-carotene, the lowest solvent consumption for the three solvent systems 2-MeTHF + (ethanol or 1-butanol) + water and CPME + 1-butanol + water was obtained for the experiment that provided the highest β-carotene yield. The lowest solvent consumption to obtain 1 mg β-carotene was achieved with the solvent system 2-MeTHF + 1-butanol + water and the experiment E1. The reason is, like for the total extraction yield, the high β-carotene yield in addition to a low mole fraction of 1-butanol for the extraction step and a short distance to the composition used in the separation step.

In conclusion, to achieve a lower solvent consumption and simultaneously high extraction yields, a solvent system composition with a high solubility of the targeted components and a higher mole fraction of water near the binodal line should be preferred for the extraction step, and for the separation step, a system composition within the biphasic region near the extraction step composition but sufficiently far from the binodal line to ensure easier phase separation should be selected.

In addition to the above criteria, the solvent’s cost and recovery should be considered when selecting biobased and green solvents for the extraction of β-carotene and lipids from *R. toruloides*. Hence, a more profound assessment of the energy costs for target component recovery and subsequent solvent recycling is needed, which presents an essential criterion to be evaluated when selecting biobased and green solvent systems.

### 3.4 Comparison with Conventional Extraction Methods

**Figure 7:**
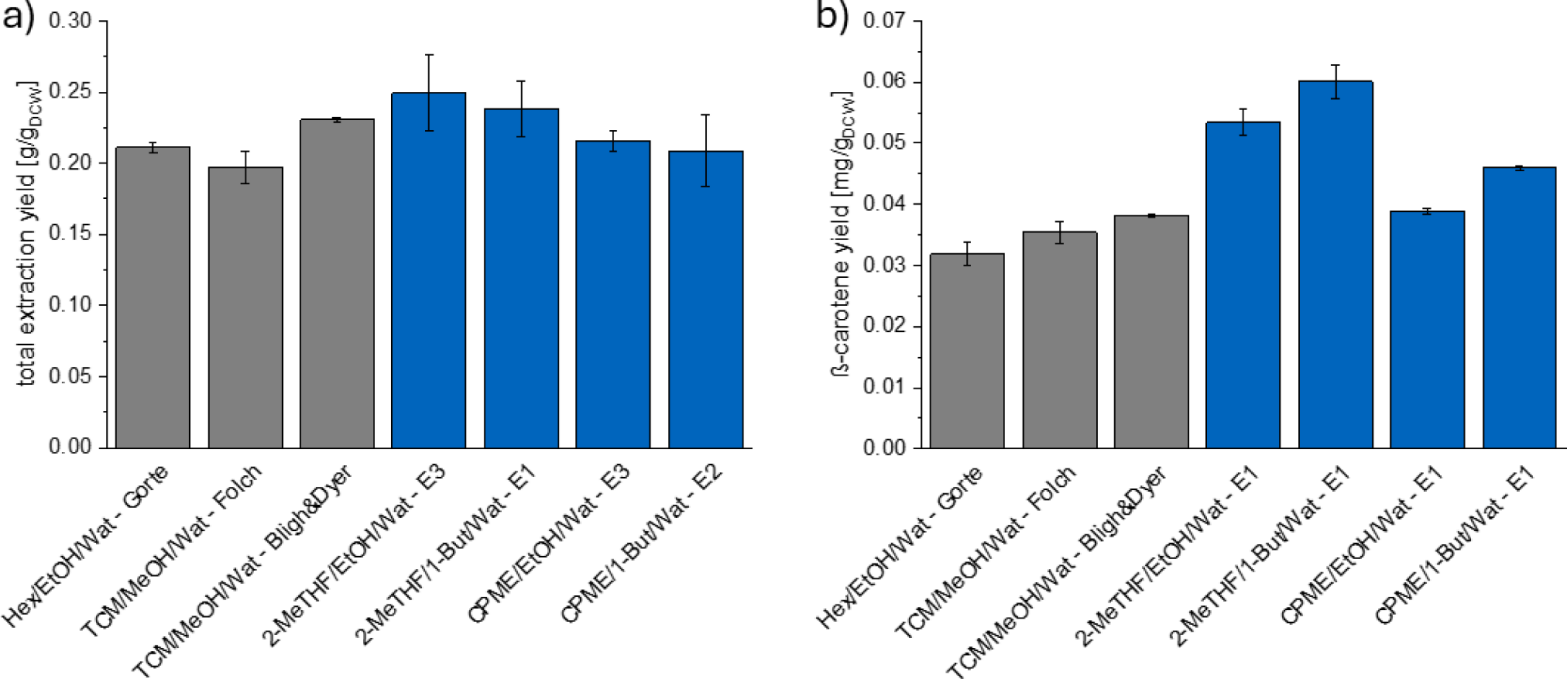
Comparison of green and biobased solvent systems (blue colored bars) with conventional solvent systems (gray colored bars) for total extraction yield (a) and β-carotene yield (b).

To assess the efficacy of the presented green and biobased solvent systems for the extraction of hydrophobic components, the extraction yields obtained with the studied green and biobased solvent systems were compared with those of the conventional solvent systems used by Folch,^12^ Bligh and Dyer,^13^ and Gorte et al.^14^ The extractions were performed, as described by the authors, and the results for the total extraction yield and β-carotene yield are presented in Figure 7. For the comparison, the system compositions of the four green and biobased solvent systems studied with the highest extraction yields were used (Figures 5 and 6).

As seen in Figure 7, the total extraction yields achieved with the green and biobased solvent systems are comparable to those obtained with the conventional solvent systems. The lowest total extraction yield was obtained with 0.20 g/g_DCW_ with the solvent system chloroform + methanol + water (Folch method). The highest total extraction yield was attained with the green and biobased solvent system 2-MeTHF + ethanol + water with 0.25 g/g_DCW_, and the yield was significantly higher than the yield achieved with the Folch method but not significantly higher than those obtained with chloroform + methanol + water (Bligh and Dyer method; 0.23 g/g_DCW_) and hexane + ethanol + water (Gorte method; 0.21 g/g_DCW_).

Regarding the β-carotene yield, the yields achieved with the green and biobased solvent systems 2-MeTHF + (ethanol or 1-butanol) + water and CPME + 1-butanol + water were significantly higher than the yields attained for all conventional solvent systems. It must be noted that the methods with conventional solvent systems were designed for the extraction of lipids from wet substances, which might explain the lower β-carotene yields. Furthermore, as shown in Figure 4, the higher the solubility of β-carotene, the bigger the mole fraction of the nonpolar solvent of the solvent systems. Due to the small single-phase region of the solvent system hexane + ethanol + water,^6^ a solvent system composition with a high mole fraction of hexane for the extraction step is not possible.

In summary, the green and biobased solvent systems achieved similar total extraction yields and higher β-carotene yields compared to the conventional solvent systems. Therefore, these solvent systems present a viable alternative to replace harmful solvent systems for the extraction of hydrophobic components from wet biomass.

### 3.5 Fatty Acid Methyl Ester (FAME) Profile of the Lipids from *R. toruloides*

A comparison of the FAME composition of the extracts from the extraction experiments, consisting of the extraction and separation steps with different system compositions for the extraction step (E1–E3) and the same composition for the separation step of all solvent systems, is shown in Figure S2, and the values are presented in Table S9.

The most prevalent fatty acids in all extracts were palmitic/oleic acid (C16:0/C16:1) and stearic/oleic acid (C18:0/C18:1), with an average of 23.18% and 60.03% of the total amount of FAME. These findings were similar to the composition reported by Jin et al.^44^ (C16:0/C16:1:26.1% and C18:0/C18:1:64.3%) and Saran et al.^45^ (C16:0/C16:1:24.38% and C18:0/C18:1:56.02%). The solvent system and system composition did not influence the total extraction yields, as seen in Figure 5, as well as on the FAME lipid profile (Figure S2).

## 4 Conclusions

The commonly used two-step process for extracting lipids and other hydrophobic components from wet biomass is realized by using biphasic liquid systems composed of conventional organic solvents. In this work, a sustainable method for extracting β-carotene and lipids from the oleaginous yeast *R. toruloides* with green and biobased solvent systems 2-methyl tetrahydrofuran (2-MeTHF) + (ethanol or 1-butanol) + water and cyclopentyl-methyl-ether (CPME) + (ethanol or 1-butanol) + water to replace harmful conventional solvent systems was investigated from a process design perspective. In particular, the influence of the system compositions used in the two steps of the process on the extraction yield and solvent consumption was critically evaluated. To gain a better understanding of the impact of the solubility of β-carotene in the solvent system composition selected for the extraction step on the extraction yields, COSMO-RS was applied to predict the β-carotene solubility within the complete single-phase regions of the four green and biobased solvent systems. Good qualitative predictions were achieved, and regions with high solubility of β-carotene within the single-phase region (rich in the nonpolar solvents 2-MeTHF and CPME) were determined.

It was found that the selection of three different solvent system compositions with different β-carotene solubilities for the extraction step had no big influence on the total extraction yield. In contrast, the extraction yield of β-carotene could be significantly increased by selecting a solvent system composition for the extraction step, in which β-carotene had a high solubility for three of the four investigated solvent systems. For the lipids, no significant difference in the extraction yield was observed in the solvent systems selected for the experimental study, except for CPME + ethanol + water. Furthermore, it was shown that the selection of solvent system compositions for both the extraction and separation steps influences the achieved extraction yield of β-carotene and solvent consumption.

To accomplish an extraction process with high extraction yields and low solvent consumption, a solvent system composition with a high mole fraction of the nonpolar solvent close to the binodal line should be selected for the extraction step, and a composition within the biphasic region close to this composition for the separation step. Additionally, the comparison with conventional extraction methods that use harmful solvents, like chloroform, showed that the presented green and biobased solvent systems achieved similar total extraction yields and even higher β-carotene yields and hence are suitable alternatives. This work highlights the successful application of green and biobased solvent systems for extracting biotechnologically produced hydrophobic components from wet biomass.

## Supporting Information Available

FAME mix composition; solvent system compositions for experimental solubility measurements; β-carotene loss during measurements; dry cell weight determination; extraction of hydrophobic components; solvent system compositions for extraction and separation step; FAME composition determination; statistical analysis; reference point deviation; pure solvents calculated β-carotene solubility; β-carotene solubility in solvent system compositions selected for extraction point; solvent consumption determination, FAME compositions (PDF)
