# Supplementary material for: Evaluation of Green and Biobased Solvent Systems for the Extraction of β-Carotene and Lipids from Rhodosporidium toruloides
Source: ACS Omega. 2025 Jan 27;10(4):4132–42. doi: 10.1021/acsomega.4c10851 (PMC11800165; doi:10.1021/acsomega.4c10851)
Supplement: Supplementary file 1 — ao4c10851_si_001.pdf [file ao4c10851_si_001.pdf]

# Supporting Information

## Evaluation of Green and Biobased Solvent Systems for the Extraction of β-Carotene and Lipids from *Rhodosporidium toruloides*

*Vanessa Buchweitz, Kilian Dauti, Ahmad Alhadid, and Mirjana Minceva*

Biothermodynamics, TUM School of Life Sciences, Technical University of Munich, 85354 Freising, Germany

* Corresponding author: Email: mirjana.minceva@tum.de, Phone number: +49 8161716170

Number of pages: 14

Number of figures: 4

Number of tables: 9

Number of equations: 5

**Table S1.** FAME Mix (C8 – C24) composition.

| Component | CAS Reg. No. | Raw Material Purity % |
|---|---|---|
| Methyl Octanoate (C8:0) | 111-11-5 | 99.9 |
| Methyl decanoate (C10:0) | 110-42-9 | 99.9 |
| Methyl laurate (C12:0) | 111-82-0 | 99.5 |
| Methyl myristate (C14:0) | 124-10-7 | 100.0 |
| Methyl palmitate (C16:0) | 112-39-0 | 99.0 |
| Methyl palmitoleate (C16:1) | 112-39-0 | 100.0 |
| Methyl stearate (C18:0) | 112-61-8 | 99.7 |
| Methyl cis-9 oleate (C18:1) | 112-61-8 | 99.6 |
| Methyl linoleate (C18:2) | 112-63-0 | 100.0 |
| Methyl arachidiate (C20:0) | 1120-28-1 | 100.0 |
| Methyl linolenate (C18:3) | 301-00-8 | 99.0 |
| Methyl behenate (C22:0) | 929-77-1 | 99.0 |
| Methyl erucate (C22:1) | 1120-34-9 | 99.4 |
| Methyl lignocerate (C24:0) | 2442-49-1 | 100.0 |

**Table S2.** Solvent system compositions for experimental solubility measurements for the systems 2-MeTHF (1) + (ethanol or 1-butanol) (2) + water (3) in mole fractions.

| $x_1$ | $x_2$ | $x_3$ |
|---|---|---|
| 2-MeTHF (1) + ethanol (2) + water (3) | | |
| 0.10 | 0.40 | 0.50 |
| 0.15 | 0.70 | 0.15 |
| 0.20 | 0.20 | 0.60 |
| 0.30 | 0.40 | 0.30 |
| 0.35 | 0.35 | 0.30 |
| 0.40 | 0.20 | 0.40 |
| 0.40 | 0.40 | 0.20 |
| 0.40 | 0.50 | 0.10 |
| 0.50 | 0.30 | 0.20 |
| 0.60 | 0.20 | 0.20 |
| 2-MeTHF (1) + 1-butanol (2) + water (3) | | |
| 0.10 | 0.50 | 0.40 |
| 0.15 | 0.70 | 0.15 |
| 0.30 | 0.40 | 0.30 |
| 0.40 | 0.40 | 0.20 |
| 0.40 | 0.50 | 0.10 |
| 0.50 | 0.20 | 0.30 |
| 0.50 | 0.30 | 0.20 |

**Table S3.** Solvent system compositions for experimental solubility measurements for the systems CPME (1) + (ethanol or 1-butanol) (2) + water (3) in mole fractions.

| $x_1$ | $x_2$ | $x_3$ |
|---|---|---|
| CPME (1) + ethanol (2) + water (3) | | |
| 0.10 | 0.50 | 0.40 |
| 0.10 | 0.30 | 0.60 |
| 0.15 | 0.70 | 0.15 |
| 0.30 | 0.40 | 0.30 |
| 0.35 | 0.35 | 0.30 |
| 0.40 | 0.40 | 0.20 |
| 0.40 | 0.50 | 0.10 |
| 0.50 | 0.30 | 0.20 |
| CPME (1) + 1-butanol (2) + water (3) | | |
| 0.10 | 0.60 | 0.30 |
| 0.15 | 0.70 | 0.15 |
| 0.30 | 0.40 | 0.30 |
| 0.40 | 0.40 | 0.20 |
| 0.40 | 0.50 | 0.10 |
| 0.50 | 0.30 | 0.20 |
| 0.65 | 0.20 | 0.15 |

**Table S4.** Mean loss of β-carotene during solubility measurements after 270 min.

| Solvent/Solvent system | Mean β-carotene loss after 270 min [%] |
|---|---|
| Ethanol | 32.83 |
| 1-butanol | 34.52 |
| 2-MeTHF | 13.10 |
| CPME | 28.66 |
| 2-MeTHF + ethanol + water | 22.00 |
| 2-MeTHF + 1-butanol + water | 25.07 |
| CPME + ethanol + water | 30.60 |
| CPME + 1-butanol + water | 27.03 |

**Dry cell weight determination**

5 ml of the unlysed biomass was centrifuged, and the supernatant was removed. Afterward, the pellet was washed with water and lyophilized with a freeze drier (Alpha 3-4 LSCbasic, Martin Christ Gefriertrocknungsanlagen GmbH, Osterode am Harz, Germany) for 24h. The samples were weighed back, and the dry cell weight (DCW) was determined gravimetrically.

**Extraction of hydrophobic components from wet biomass**

The extraction is performed in the single-phase region of the solvent systems phase diagram to disrupt the strong interactions between lipids and cell biopolymers and ensure sufficient access of the non-polar solvent to the hydrophobic target components. In the extraction step (E), see Figure S1, carried out within the single phase region, the hydrophobic and hydrophilic components of the biomass are extracted. In the following separation step (S) within the biphasic region, the hydrophobic target components (solvent 1-rich phase ●) are separated from the hydrophilic components (water-rich phase ●).

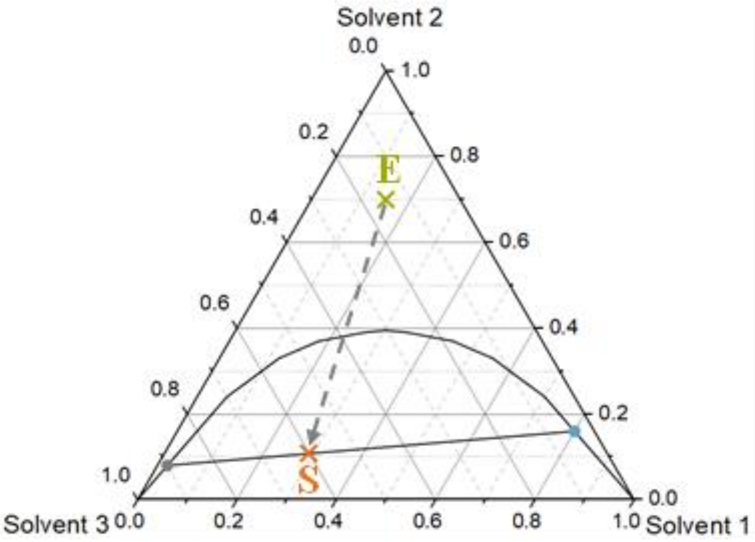


**Figure S1.** Exemplary ternary diagram in mole fractions with the extraction step (E) and the separation step (S)

To 1 g of lysed biomass of *R. toruloides*, considered as water, corresponding masses of solvent 1 (2-MeTHF or CPME) and solvent 2 (ethanol or 1-butanol) to attain the solvent composition for the extraction step (E) were added in a vial. The vials with the mixture were turned upside-down 20 times, wrapped in aluminum foil, and then shaken for 3 hours at room temperature with an overhead mixer (Multi Bio RS-24, Kisker Biotech GmbH & Co. KG, Germany). Afterward, the samples were centrifuged for 10 min at 4800 rpm in a temperature-controlled centrifuge (Sigma 3-16KL, rotor 11133, Sigma Laborzentrifugen GmbH, Germany) to remove cell debris. For the separation step, 5 g of the supernatant was transferred to a new vial, and corresponding masses of water and solvent 1 (2-MeTHF or CPME) were added to obtain the solvent system composition of the separation step (S) to achieve phase separation. After that, the samples were shaken with an overhead mixer for 3 min and centrifuged for 10 min at 4800 rpm. Then, 5 g of the upper phase was taken and evaporated in a rotary vacuum concentrator (RVC 2-25 CDplus, Martin Christ Gefriertrocknungsanlagen GmbH, Germany) at 30 °C and 40 mbar to obtain the final extract.

Each extraction experiment, consisting of extraction step (E) and separation step (S), was performed three times, and blank extractions without biomass were used to exclude artifacts.

The total extraction yield was calculated using the following formulas:

$$total\ extraction\ yield\ \left[\frac{\mathrm{g_{extract}}}{\mathrm{g_{DCW}}}\right] = \frac{m_{extract}}{m_{biomass} \cdot DCW} \tag{S1}$$

$$m_{extract}[\mathrm{g}] = m_{crude\ extract} - m_{blank} \tag{S2}$$

$$\beta - carotene\ yield\ \left[\frac{\mathrm{mg_{\beta-carotene}}}{\mathrm{g_{DCW}}}\right] = \frac{m_{\beta-carotene}}{m_{biomass} \cdot DCW} \tag{S3}$$

where $m_{biomass}$ is the mass of biomass that was used for the extraction process; $DCW$ is the dry cell weight of the biomass; $m_{crude\ extract}$ is the mass of extract obtained in the extraction process; $m_{blank}$ is the mass obtained in the extraction without biomass (artifacts); $m_{extract}$ is the extracted mass without artifacts; $m_{\beta-carotene}$ is the mass of β-carotene obtained during the extraction process, respectively.

**Table S5.** Solvent system composition in mole fraction for the extraction and separation step and the corresponding solid-liquid ratio for the extraction step for the solvent systems 2-MeTHF + (ethanol or 1-butanol) + water, CPME + (ethanol or 1-butanol) + water.

| Step | Experiment | $x_1$ | $x_2$ | $x_3$ | Solid-liquid ratio [mg $g^{-1}$] |
|---|---|---|---|---|---|
| 2-MeTHF (1) + ethanol (2) + water (3) | | | | | |
| | 1 | 0.70 | 0.10 | 0.20 | 8.06 |
| Extraction | 2 | 0.50 | 0.15 | 0.35 | 17.13 |
| | 3 | 0.35 | 0.30 | 0.35 | 19.23 |
| Separation | 1-3 | 0.36 | 0.03 | 0.61 | |
| 2-MeTHF (1) + 1-butanol (2) + water (3) | | | | | |
| | 1 | 0.60 | 0.25 | 0.15 | 7.74 |
| Extraction | 2 | 0.45 | 0.35 | 0.20 | 12.97 |
| | 3 | 0.30 | 0.30 | 0.40 | 19.94 |
| Separation | 1-3 | 0.42 | 0.06 | 0.52 | |
| CPME (1) + ethanol (2) + water (3) | | | | | |
| | 1 | 0.70 | 0.10 | 0.20 | 5.58 |
| Extraction | 2 | 0.55 | 0.15 | 0.30 | 8.53 |
| | 3 | 0.35 | 0.35 | 0.30 | 14.60 |
| Separation | 1-3 | 0.34 | 0.11 | 0.55 | |
| CPME (1) + 1-butanol (2) + water (3) | | | | | |
| | 1 | 0.60 | 0.25 | 0.15 | 5.10 |
| Extraction | 2 | 0.40 | 0.40 | 0.20 | 7.52 |
| | 3 | 0.15 | 0.50 | 0.35 | 16.56 |
| Separation | 1-3 | 0.36 | 0.07 | 0.57 | |

**Determination of the composition of Fatty Acid Methyl Esters (FAME)**

To determine the composition of the fatty acid methyl esters in the extract obtained with the two-step process, the extracts were dissolved in the corresponding mass of hexane to reach a concentration of 20-50 mg/ml. Then 6 ml of a methanol-hydrochloric acid mixture (11:1; v/v%) was added to 200 μl of the dissolved extracts for transesterification and the samples were heated at 90 °C in a block thermostat (HB-48 WiseTherm®, witeg Labortechnik GmbH, Germany) for 1 h. Afterwards, 1.7 ml of hexane and 2 ml of water were added to achieve a phase separation, and the upper phase, which contained the fatty acid methyl esters, was analyzed. Tridecanoic acid was used as the internal standard.

The compositions of the fatty acid methyl esters were analyzed with a 6890N Network GC System and 7683 Series Injector coupled with a 5973 Network Mass Selective Detector (Agilent Technologies, Inc, USA). A RTx®-Wax column (30 m length, 0.25 inner diameter, 0.5 μm film thickness, Restek, Bellefonte, PA, USA) was used in split mode, with a split ratio of 1:7.5 and an injection port temperature of 473.15 K and injection volume of 1 μl. As a carrier gas Helium was used at a linear velocity-controlled flow of 1.0 ml/min. The initial column temperature was set for 2 min at 353.15 K and then increased with a linear gradient of 7 K/min until 453 K. This column temperature was held for 10 min followed by an increase until 503.15 K with a linear gradient of 1 K/min and was held for another 10 min. A calibration curve of pure fatty acids methyl esters was used to determine the lipid profile.

**Statistical analysis**

All experimental results are presented as mean ± standard deviation, and one-way analysis of variance with Turkey's post-hoc tests were performed with the OriginPro 2023 software (OriginLab Corporation, USA) with a statistically significant of $p < 0.05$.

**Table S6.** Mean standard deviation of all corrected β-carotene solubilities within the single phase region obtained with different reference points for each solvent system

| Solvent system | Solvent system composition of the selected reference point | Mean standard deviation [mg/g] |
|---|---|---|
| 2-MeTHF + ethanol + water | 0.40/0.20/0.40 | 0.02 |
| 2-MeTHF + 1-butanol + water | 0.15/0.70/0.15 | 0.07 |
| CPME + ethanol + water | 0.35/0.35/0.30 | 0.05 |
| CPME + 1-butanol + water | 0.40/0.40/0.20 | 0.04 |

**Table S7.** Calculated β-carotene solubility in the pure solvents

| Solvent system | Calculated solubility [mg/g] | Measured solublity [mg/g] |
|---|---|---|
| Ethanol | 0.16 | 0.18 |
| 1-butanol | 0.38 | 0.25 |
| 2-MeTHF | 15.05 | 10.35 |
| CPME | 12.95 | 9.52 |

**Table S8.** Predicted β-carotene solubility in the solvent system compositions used for the extraction point.

| Solvent System | Extraction step (mol%) | β-carotene solubility [mg/g] |
|---|---|---|
| | E1 (0.70/0.10/0.20) | 4.39 |
| 2-MeTHF + ethanol + water | E2 (0.50/0.15/0.35) | 0.84 |
| | E3 (0.35/0.30/0.35) | 0.42 |
| | E1 (0.70/0.10/0.20) | 3.55 |
| 2-MeTHF + 1-butanol + water | E2 (0.55/0.15/0.30) | 1.26 |
| | E3 (0.30/0.30/0.40) | 0.47 |
| | E1 (0.60/0.25/0.15) | 4.11 |
| CPME + ethanol + water | E2 (0.45/0.35/0.20) | 1.52 |
| | E3 (0.35/0.35/0.30) | 0.52 |
| | E1 (0.60/0.25/0.15) | 2.41 |
| CPME + 1-butanol + water | E2 (0.40/0.40/0.20) | 1.00 |
| | E3 (0.15/0.50/0.35) | 0.49 |

**Determination of the solvent consumption**

The solvent consumption necessary to obtain the extraction for the extraction experiments (E1-E3) for the solvent systems 2-MeTHF + (ethanol or 1-butanol) + water and CPME + (ethanol or 1-butanol) + water was calculated with the following formulas:

$$solvent\ consumption\ (total\ extract)\ \left[\frac{\mathrm{kg_{solvent}}}{\mathrm{g_{extract}}}\right] = \frac{m_{2-MeTHF}+m_{ethanol}+m_{water}}{total\ extraction\ yield\ \left[\frac{\mathrm{g_{extract}}}{\mathrm{g_{DCW}}}\right]} \tag{S4}$$

$$solvent\ consumption\ (\beta-carotene)\ \left[\frac{\mathrm{kg_{solvent}}}{\mathrm{mg_{\beta-carotene}}}\right] = \frac{m_{2-MeTHF}+m_{ethanol}+m_{water}}{\beta-carotene\ yield\ \left[\frac{\mathrm{mg_{\beta-carotene}}}{\mathrm{g_{DCW}}}\right]} \tag{S5}$$

were $m_{2-MeTHF}$, $m_{ethanol}$, and $m_{water}$ are the masses of 2-MeTHF, ethanol and water added in both the extraction and separation step; $total\ extraction\ yield\ \left[\frac{\mathrm{g_{extract}}}{\mathrm{g_{DCW}}}\right]$ is the total extraction

yield calculated with Eq. S1 and $\beta - carotene\ yield\ \left[\frac{mg_{\beta-carotene}}{g_{DCW}}\right]$ the β-carotene yield calculated with Eq. S3 for each extraction experiment (E1-E3) for all four solvent systems.

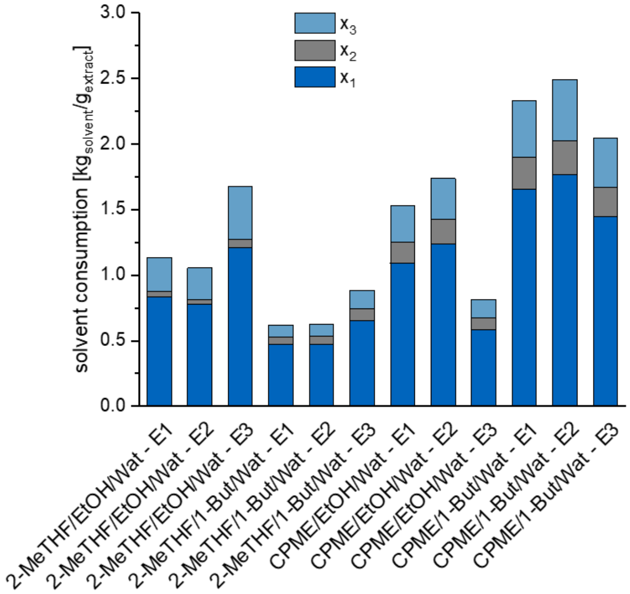


**Figure S2.** Solvent consumption for 1 g total extract for the extraction experiments (E1-E3) for the solvent systems 2-MeTHF ($x_1$) + (ethanol or 1-butanol) ($x_2$) + water ($x_3$) and CPME ($x_1$) + (ethanol or 1-butanol) ($x_2$) + water ($x_3$).

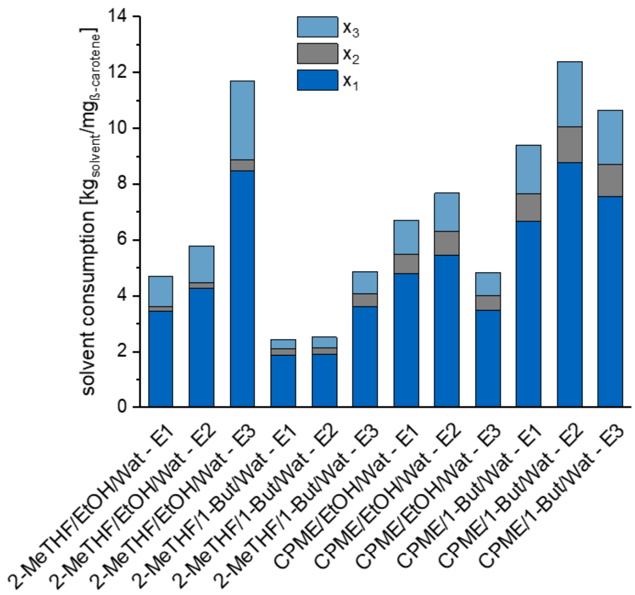


**Figure S3.** Solvent consumption for 1 mg β-carotene for the extraction experiments (E1-E3) for the solvent systems 2-MeTHF ($x_1$) + (ethanol or 1-butanol) ($x_2$) + water ($x_3$) and CPME ($x_1$) + (ethanol or 1-butanol) ($x_2$) + water ($x_3$).

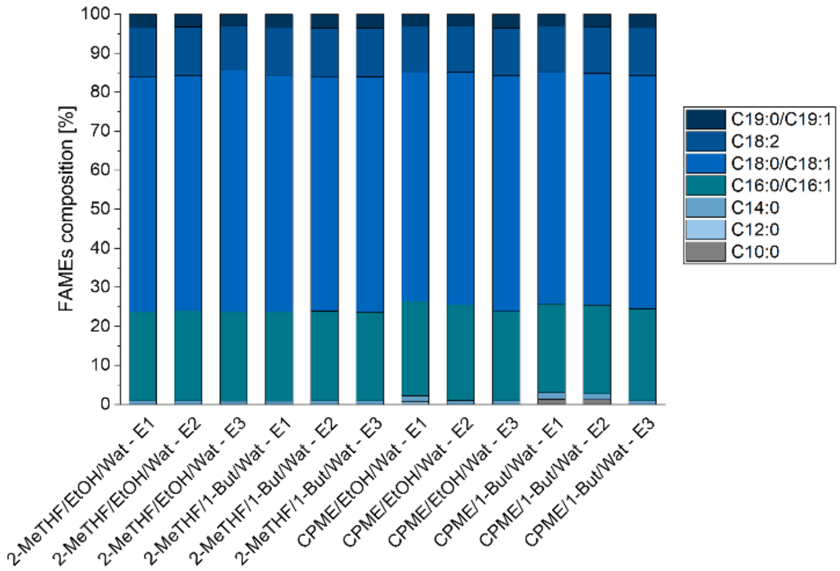


**Figure S4.** Comparison of the FAMEs composition of the extracts for all extraction experiments (E1-E3) with different system compositions for the extraction step for the solvent systems 2-MeTHF + (ethanol or 1-butanol) + water and CPME + (ethanol or 1-butanol) + water.

**Table S9**. FAME composition [%] of all extracts from all extraction experiments (E1-E3) for the solvent systems 2-MeTHF + (ethanol or 1-butanol) + water and CPME + (ethanol or 1-butanol) + water.

| Fatty acid | 2-MeTHF + EtOH + Wat | | | 2-MeTHF + 1-But + Wat | | | CPME + EtOH + Wat | | | CPME + 1-But + Wat | | |
|---|---|---|---|---|---|---|---|---|---|---|---|---|
| | E1 | E2 | E3 | E1 | E2 | E3 | E1 | E2 | E3 | E1 | E2 | E3 |
| C10:0 | 0 | 0 | 0 | 0 | 0 | 0 | 0 | 0 | 0 | 1.33 | 1.23 | 0 |
| C12:0 | 0 | 0 | 0 | 0 | 0 | 0 | 0.74 | 0 | 0 | 0 | 0 | 0 |
| C14:0 | 0.88 | 0.89 | 0.81 | 0.86 | 0.88 | 0.90 | 1.50 | 0.98 | 0.89 | 1.66 | 1.49 | 0.91 |
| C16:0/C16:1 | 22.82 | 23.15 | 22.61 | 22.84 | 23.00 | 22.77 | 24.21 | 24.55 | 23.26 | 22.76 | 22.77 | 23.52 |
| C18:0/C18:1 | 60.19 | 60.19 | 60.37 | 60.47 | 60.03 | 60.29 | 58.79 | 59.66 | 59.27 | 59.30 | 59.36 | 59.81 |
| C18:2 | 12.70 | 12.45 | 12.68 | 12.41 | 12.60 | 12.48 | 11.62 | 11.67 | 12.90 | 11.87 | 11.99 | 12.32 |
| C19:0/C19:1 | 3.41 | 3.32 | 3.53 | 3.42 | 3.49 | 3.56 | 3.14 | 3.14 | 3.68 | 3.08 | 3.16 | 3.44 |
